# Leveraging the Hyperledger Fabric for Enhancing the Efficacy of Clinical Decision Support Systems

**DOI:** 10.30953/bhty.v4.154

**Published:** 2021-02-17

**Authors:** Ramya Gangula, Sri Varun Thalla, Ijeoma Ikedum, Chineze Okpala, Sweta Sneha

**Affiliations:** Department of Information Systems and Security, Kennesaw State University, Kennesaw, Georgia

**Keywords:** CDSS, Hyperledger Fabric, blockchain, interoperability, alert fatigue, patient outcome

## Abstract

Adopting and implementing the Clinical Decision Support System (CDSS) technology is a critical element in an effort to improve national quality initiatives and evidence-based practice at the point of care. CDSS is envisioned to be a potential solution to many current challenges in the healthcare sphere, which includes information overload, practice improvement, eliminating treatment errors, and reducing medical consultation costs. However, the CDSS did not manage to achieve these goals to the desired levels and provide context-appropriate alerts, although integrated with the electronic health records (EHRs) (1). Clinical decision support alerts can save lives, but frequent ones can cause increased cognitive burden to clinicians, worsen alert fatigue, and increase the duplication of tests. This ultimately increases health care costs without refining patient outcomes. Studies show that 49–96% of clinical alerts are ignored, raising questions about the effectiveness of CDSS (1). Blockchain, a decentralized, distributed digital ledger that contains a plethora of continuously updated, time-stamped, and highly encrypted virtual record, can be a key to addressing these challenges (2). The blockchain technology if integrated with the CDSS can serve as a potential solution to eliminating current drawbacks with CDSS (3). This article addresses the most significant and chronic problems facing the successful implementation of CDSS and how leveraging the Hyperledger Fabric can alleviate the clinical alert fatigue and reduce physician’s burnout using patient-specific information. The proposed architecture framework for this study is designed to equip the CDSS with overall patient information at the point of care. This then empowers the physicians with the blockchain-integrated CDSS, which holds the potential to reduce clinician’s cognitive burden, medical errors, and costs and ultimately enhance patient outcomes. The research study broadly discusses how the blockchain technology can be a potential solution, reasons for selecting the Hyperledger Fabric, and elaborates on how the Hyperledger Fabric can be leveraged to enhance the efficacy of CDSS.

Clinical decision support systems (CDSS) are computer-based applications that analyze and intelligently filter data within electronic health records (EHRs) to assist providers, clinicians, administrative staff, and patients at the point of care. They comprise of several tools that filter through the tons of data and provide suggestions on the next steps in treatment (4). The CDSS tools that aid in decision- making include ‘computerized alerts and reminders for providers and patients; clinical guidelines; condition-specific order sets; focused patient data reports and summaries; documentation templates; diagnostic support, and contextually relevant reference information, among other tools (5)’. The aims of integrating CDSS into the foundation of EHR were to improve the quality of care by avoiding medical errors, such as adverse drug events and incorrect dosage prescriptions, and to lower health care expenses and enhance patient outcomes (4, 7).

The integration of CDSS enables in combining prescription information with patient information and provides alerts to patients when there are drug-drug interactions, drug-allergy contraindications, and other critical situations. In addition, it assists the providers in ordering medications, lab and imaging tests (8).

## Drawbacks of CDSS

While CDSS alerts are supposed to enhance patient safety by being the solution to adverse drug events and inappropriate dosage prescription, they are not functionally as effective as they ought to be. Also, alert fatigue in clinicians has been consistently on ECRI’s (Emergency Care Research Institute) top ten health technology hazards (6).

The lack of efficacy of CDSS is due to alert fatigue, resulting from the repeated incurrence of irrelevant pop-ups or warnings related to the treatment. Alert fatigue is because CDSS can only access the patient information silos in the current EHR (1, 9). When integrated into the EHR, CDSS provides alert based on limited patient information. This results in generating inappropriate and irrelevant alerts that are neither patient specific nor context specific to the situation. The plethora of irrelevant alerts results in a cognitive overload on the providers, leading to desensitization, physician burnout and eventually medical errors (10).

Furthermore, CDSS is aimed to assist providers in arriving at diagnosis by providing suggestions regarding the treatment plan (1, 8). It aids them in making decisions regarding further diagnostic tests and procedures. As CDSS integrates medication information with inadequate patient data, it sometimes leads to ordering (11) duplicate diagnostic tests and inappropriate prescriptions, resulting in an enhanced patient risk and health expenditure (12).

Although the purpose of CDSS is to enable better patient outcomes and reduce the burden on providers, this purpose is not being served adequately (1, 9). This article intends to address this issue by leveraging the blockchain technology to improve CDSS functionality.

## Blockchain technology

Blockchain is a decentralized, distributed ledger technology (DLT), with members in the network denoted as nodes. The transactions made among them are recorded by every node in the network into a distributed ledger (11). All nodes validate the information to be appended to the ledger, and a consensus protocol ensures that the nodes agree to a unique order in which entries are appended. Once an agreement is reached, data are permanently recorded in sequential, append-only, tamper-evident blocks to the ledger (13).

**Fig. 1:**
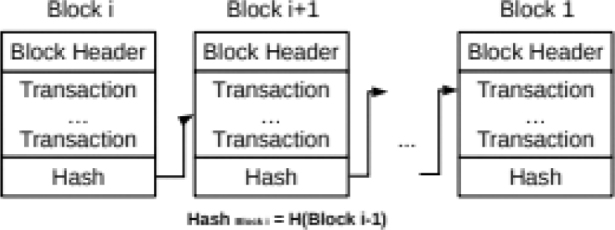
Blockchain structure (13).

All the confirmed and validated transaction blocks are hash linked from the origin block to the most current block represented in the Figure 1, making the ledger a chain of linked blocks (blockchain) and also harder to change or delete data in any block without network consensus. This property is referred to an immutability of ledger (11). The signature or hash consists of a cryptographically generated sequence of letters and numbers of a defined length that uniquely identifies any (defined and acceptable) digital entity (14). Each record in the Blockchain includes precise information about when it was created (timestamp) and the cryptographic signature of the preceding document in the chain, and the transaction data in this case health record. Immutability lends itself to trust system of any blockchain network (15).

There are two kinds of blockchain networks, which are public, permission-less blockchains and private, permissioned blockchains. The principal distinction between public and private blockchain platforms is that anyone can join the public platforms, while only authenticated users can join a private, permissioned blockchain framework. The popular examples of permission-less blockchain platforms include Bitcoin, Ethereum, and Litecoin, and those of private, permissioned blockchains include Hyperledger, Multichain, R3 Corda, and so on (11).

## Research objective

With the limitations of CDSS above elucidated, this study aims to propose a conceptual model to enhance functionality of CDSS in order to improve patient outcomes and reduce cognitive burden on providers. The conceptual model aims to be formulated leveraging a private, permissioned blockchain framework, Hyperledger Fabric, to facilitate interoperability of patient health information and ensure its safety and privacy while being transacted in between healthcare systems.

The following sections of the article provide a background on the Hyperledger Fabric and its components, elucidating the conceptual model, cite the reasons for selecting Hyperledger Fabric over other private, permissioned blockchain platforms, and demonstrate how the proposed model intends to work in a real-world scenario.

## Background

### Hyperledger Fabric

Hyperledger is an open-source umbrella project, hosting several distributed ledger frameworks, tools, and libraries. It is a business-driven blockchain framework, which is built to help business organizations to transact through a private blockchain network by The Linux Foundation along with other collaborators (16).

Hyperledger Fabric is one of the Hyperledger frameworks, which is a private, permissioned blockchain in which organizations can participate in data sharing (17).

### Components of the Hyperledger Fabric

#### Membership service provider

Membership service provider (MSP) serves as a certificate authority, trusted by all the members in the network. This entity validates the identity of an organization and authorizes its participation in the network (17) by issuing it a root certificate to become a member in the Hyperledger Fabric framework, and hence, permissioned blockchain framework. There can be single or multiple membership service providers in a Hyperledger Fabric network, and they are pluggable function, which can be plugged-in by the members instead of building their own (18).

#### Nodes

Members in the Hyperledger Fabric communicate with each other through their nodes. Each member has three different kinds of nodes serving different functions.

*Client node.* This is the end user or application, who or which needs information and initiates the transaction (19).

*Ordering service node*. This pluggable node is responsible for ordering the transactions, packaging them as blocks, and disseminating these blocks to the peers in the network. At least one of the members in the network must be an ordering service node (16).

*Peers*. The peer nodes with chain code or smart contract installed on their machines endorse the transaction requests received based on the endorsement policy and validate the transactions before committing to the ledger (20).

#### Smart contract

The smart contract is the business logic algorithm that is mutually agreed upon by all the organizations in the network. It is installed on all the endorsing peers on the network, and when executed it records and distributes conditional transactions in an immutable manner, instituting trust among the participants of the transaction (11, 15).

#### Ledger

Ledger in the fabric consists of two components: world state and transaction log (17).

*World state*. It describes the current state of the ledger at that point and holds all the latest transactions or assets.

*Transaction log*. It holds all the previous transactions, leading to the current transaction in the world state.

The data in the ledger are in the form of blocks that are cryptographically linked to each other (19).

**Fig. 2:**
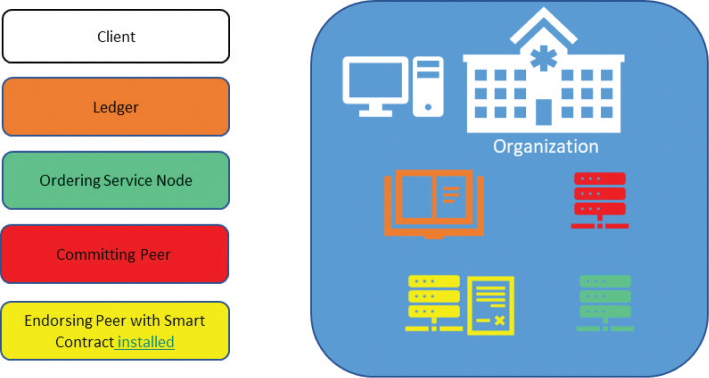
Representation of a member with nodes and ledger in a Hyperledger Fabric.

Figure 2 shows three types of nodes, that is, client node, ordering service node, and peer nodes (endorsing peer with the chain code on its machine and committing peer) and a decentralized ledger in a single channel in a Hyperledger Fabric framework.

## Methods

### Conceptual model – Hyperledger Fabric framework

**Fig. 3:**
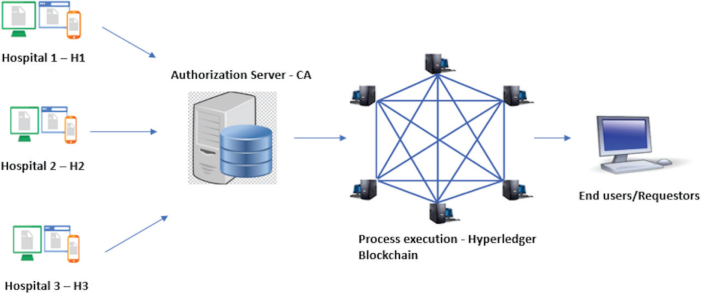
Proposed model for exchange of information between nodes in a Hyperledger network.

In this section, we elucidate the overall picture of how health information can be exchanged among healthcare organizations, employing a Hyperledger Fabric framework using a conceptual model illustrated in Fig. 3.

Let us suppose H1, H2, and H3 are three independent healthcare organizations that are leveraging the Hyperledger Fabric to exchange information between them.

1. The MSP authenticates all the organizations who are willing to transact using the Fabric framework. One of the organizations acts a consortium leader and creates a channel to add other organizations to the channel.
2. Let us assume that organization ‘H3’ requests some information, and then the client node of H3 initiates a transaction invocation request and sends it to all the endorsing peers in the network.
3. The endorsing peers of H1 and H2 will receive the transaction request and simulate a transaction by executing the chain code on their machines. This results in generation of RW (Read-Write) sets, which includes the information on what could have been read or written onto the ledger had the transaction been executed. The endorsing peers now endorse or reject the transaction invocation based on the endorsement policy. If majority of the endorsement peers approve the transaction, the endorsement decision along with RW sets are sent back to the client.
4. Organization H3 through its client application now will check for the endorsement, and if it is approved, it will be forwarded to the ordering service node in the network.
5. The ordering service node verifies the endorsement of transaction, client identity, and orders and packages the information in the form of blocks in the order, which they are to be committed to the ledger. This node now disseminates the blocks of information to all the committing peers in the network.
6. The committing peers validate the transaction and commit the blocks to the ledger in the order in which they are received.

## Discussion

### Why Hyperledger Fabric?

The word ‘data breach’ sends jitters among the individuals or organizations that deal with sensitive data or information. According to the report by Ponemon Institute and Verizon Data Breach Investigations, healthcare industry is more vulnerable to data breaches than any other sector (21), and nearly 60% of the data breach incidents pertaining to health information (PHI) involved insiders (22).

Given the importance of personal health information and the need to comply with regulations [The Health Insurance Portability and Accountability Act of 1996 (HIPAA) and General Data Protection Regulation (GDPR)] in place, privacy, security, and scalability of the health information exchange are of paramount importance. While there are multiple public and private blockchain platforms that provide similar advantages in terms of information exchange and data privacy, health care data transfer requires minimal latency with heightened security. This concoction and the criticality of medical information interchange are the driving factors behind choosing the Hyperledger Fabric among the other private, permissioned blockchain platforms. Also, according to the results published in the research (23), Hyperledger Fabric demonstrated increased privacy and throughput with minimal latency compared with other private, permissioned platforms, such as ‘Quorum’, ‘Multichain’, and ‘R3 Corda’ (23). A detailed comparison amongst various private blockchain platforms (Hyperledger Fabric, Quorum, Multichain, R3 Corda) is provided by Polge et al. in terms of privacy, scalability, throughput and latency (23).

The Hyperledger Fabric is a platform for distributed ledger solutions underpinned by a modular architecture delivering high degrees of confidentiality, resiliency, flexibility, and scalability. The architecture and design of Hyperledger Fabric makes it very suitable and effective platform to exchange vulnerable information like PHI.

The Fabric with its malleable design ensures privacy and confidentiality of the transactions (24) among the members in the channel or between a subset of organizations in a sub-network. That means, in cases where members want to share an information exclusive to a group in the channel, they can do so via forming a separate channel in the framework (additional administrative overhead) or by creating a sub-network (bypasses overhead) in the existing channel.

This flexibility in designing of Fabric allows creation of private data collections between a group of members where the transaction can be endorsed, validated, and committed to the private state of the ledger by the authorized peers in a subnetwork. The private transactions are broadcasted without ordering service node via a peer-to-peer gossip protocol, thereby ensuring privacy and confidentiality of the exclusive transaction from the members outside the sub-network in the same channel (25).

The Fabric’s modular architecture and design provide ease of operation with plug-and-play features and furnish high performance, as there are specific nodes for specific purposes ensuring high transaction throughput and scalability (19, 25).

Moreover, with the Fabric being a permissioned platform, only vetted, authenticated organizations can participate and transact in the channel (25).

### How Hyperledger Fabric aids CDSS?

Let us consider a scenario, where a patient continues to visit a health care provider for a long time. The entire medical history records are stored in the EHR system (of the provider) and in the event of the patient being referred to a new specialist, the new provider would not have history on the patient’s medical condition in its entirety. In this case, the CDSS in the EHR of the specialist provider might recommend context inappropriate alerts due to incomplete patient information. This can potentially lead CDSS to fire inappropriate alerts and result in alert fatigue in physicians as well as ordering of duplicate tests and medications for the patients, thereby increasing the treatment costs (1, 8).

The problem in the given scenario is not with the core functionality of CDSS as the alerts are based on patient information available in the EHR system. If the entire patient information is made available to the EHR of specialist provider, then the CDSS can potentially provide context appropriate alerts based on the entire patient history, in turn helping the patient in diagnosis and the treatment of the disease (26). With the Hyperledger Fabric framework, the patient’s primary care provider and the specialist provider can form a channel with smart contract installed and exchange patient information, which is immutable. As all the ledgers in a given channel are identical, entire patient information is present with both the providers in the same sequential manner and the safety of the information is ensured (23, 24). This can help in enhanced performance of CDSS, reducing cognitive burden on providers and most importantly improving patient outcomes.

## Conclusion and future work

This article broadly talks about how Hyperledger serves as a potential solution to enhance interoperability, which, in turn, addresses issues related to CDSS. Blockchain has been a proven technology that other dependent lineups can be built on. This being a distributed ledger and an immutable transaction recorder, resistance to changes is natural and any updates can be traced back easily. We chose the Hyperledger Fabric and proposed a conceptual model on how information can be exchanged among independent hospitals or systems in a private and secured manner. This study starts off by listing out issues pertaining to CDSS, which were having an impact on the overall patient outcomes. This work then moves onto isolating the root cause that is, how interoperability amongst participating systems has been the core concern and how Hyperledger Fabric can be a viable solution in enhancing the fundamental functionality of CDSS. While the current article focuses on the conceptual aspects and provides a framework on how Hyperledger Fabric can aid CDSS, additional research work will be required when it comes to practical implementation, to address ground level application issues & deal with real-time scenarios.

## Data Collection

Ramya Gangula, Sri Varun Thalla, Ijeoma Ikedum, Chineze Okpala

## Data analysis and interpretation

Ramya Gangula, Sri Varun Thalla

## Drafting the article

Ramya Gangula, Sri Varun Thalla, Chineze Okpala, Ijeoma Ikedum

## Critical revision of the article

Ramya Gangula, Sri Varun Thalla, Ijeoma Ikedum, Sweta Sneha

## Final approval of the version to be published

Ramya Gangula, Sri Varun Thalla, Ijeoma Ikedum, Chineze Okpala, Sweta Sneha
